# Implementation of a clinical practice guideline for schizophrenia in a specialist mental health center: an observational study

**DOI:** 10.1186/s12913-016-1618-9

**Published:** 2016-08-11

**Authors:** Ilan Fischler, Sanaz Riahi, Melanie I. Stuckey, Philip E. Klassen

**Affiliations:** 1 Ontario Shores Centre for Mental Health Sciences, 700 Gordon Street, Whitby, ON L1N 5S9 Canada; 2 Department of Psychiatry, University of Toronto, Toronto, Canada; 3 Faculty of Health Sciences, Univeristy of Ontario Institute of Technology, Oshawa, Canada

**Keywords:** Schizophrenia, Quality improvement, Mental health, Clinical practice guidelines, Evidence-based practice, Implementation research

## Abstract

**Background:**

In mental health settings, implementation of and adherence to clinical practice guidelines (CPGs) is low. Strategies are needed to overcome barriers and facilitate successful implementation of CPGs into standard care. The goals of this study were to develop a framework for the implementation of a CPG for schizophrenia for hospitalized service users in a mental health care facility, and to monitor adherence to the guideline.

**Methods:**

An eight-step framework was developed based on project management principles: 1) the Appraisal Guideline for Research and Evaluation (AGREE) tool was used to rate and select a CPG; 2) an algorithm was created from the guideline; 3) a gap analysis identified clinical services and processes not conforming with the CPG recommendations; 4) a governance structure was created; 5) a modified Delphi process determined key outcome and process adherence metrics; 6) a project charter was developed; 7) clinical informatics ensured that systems and tools were in place to support the CPG; and 8) therapeutic services were realigned to match the requirements of the CPG within specified fiscal constraints. Percent adherence to the identified process adherence metrics was calculated before (March 2014) and for 12 months after implementation (April 2014-March 2015).

**Results:**

The National Institute of Health and Care Excellence guideline scored highest on AGREE and was used to develop the algorithm. Cognitive behavior therapy for psychosis (CBT-P), art therapy and carer assessments were identified as gaps in care. Clinical global impression – Schizophrenia score was identified as the primary service user outcome variable and antipsychotic polypharmacy, metabolic monitoring, CBT-P referral and supported employment/vocational services referral as the primary process adherence measures. Adherence to guidance for metabolic monitoring (March 2014, 76.7 %; March 2015, 81.6 %), CBT-P referral (March 2014, 6.5 %; March 2015, 11.4 %) and vocational rehabilitation referral (March 2014, 36.6 %; March 2015, 49.1 %) were increased after CPG implementation. There was an initial increase in adherence to antipsychotic monotherapy (March 2014, 53.4 %; November 2014, 62.7 %), which decreased back toward baseline (March 2015, 55.1 %).

**Conclusions:**

The eight-step framework was used to implement a CPG process, though further quality improvements initiatives may be needed to improve adherence.

## Background

Clinical practice guidelines (CPGs) are developed based on a synthesis of scientific evidence regarding the best approaches for the assessment, diagnosis and treatment of a particular clinical domain or diagnosis in order to optimize care [1]. Research examining adherence to CPGs in the mental health sector has shown mixed results [2–10], and most studies report adherence to only one component of the guideline. Results from physician surveys have identified a number of barriers to the adoption of, and adherence to, CPGs including: lack of awareness of or disagreement with guidelines; insufficient motivation to change; negative attitudes toward guidelines in general; faith in existing treatment practices; and lack of time, availability and organizational support [11, 12]. The local context has been identified as an important factor in determining the success of CPG implementation [11]. For example, organizational culture and leadership, evaluation of practices and performance feedback are important facilitators to implementation [11]. Thus, a systematic implementation of a CPG at the organizational level targeting common barriers could lead to better adherence. A recent review determined that multifaceted implementation strategies including educational materials or meetings along with reminders and coordination by a member of the healthcare team were most likely to improve adherence following CPG implementation [13].

Ontario Shores Centre for Mental Health Sciences’ (Ontario Shores) 5-year strategic plan includes the systematic implementation of CPGs with the goal of ensuring that service users and families are provided with the full complement of assessments and treatments recommended by CPGs. Ontario Shores considered CPG implementation as an opportunity to become a data driven organization, using clinical measures to drive quality improvement and improve outcomes for service users. The first CPG to be implemented at Ontario Shores was for the assessment and treatment of schizophrenia and schizoaffective disorder for hospitalized service users. It was recognized that an organization-wide strategy with strong leadership aimed at facilitating implementation while overcoming common barriers would be essential for success [11–13]. The purpose of this paper was to describe the implementation of CPGs for schizophrenia and schizoaffective disorder at Ontario Shores and to present CPG adherence data over the 12 months following implementation.

## Methods

This observational study was carried out as a corporate evaluation initiative at Ontario Shores (Whitby, Canada), a 326-bed public teaching hospital specializing in comprehensive mental health and addiction services for those with complex serious and persistent mental illness. CPG-recommended treatments were to be applied to any service user admitted to a general adult or forensic unit with a primary diagnosis of schizophrenia or schizoaffective disorder.

Two types of working groups were formed to support implementation: The Physicians representing each clinical program within the organization were appointed to the Physician-Advisory Group on the basis of interest, clinical expertise and leadership. Representatives from Clinical Informatics and Professional Practice were included in this committee. The interprofessional design working groups included the Medical Director of Clinical Informatics (a psychiatrist), representatives from Professional Practice and Decision Support, the manager and representatives from Clinical Informatics, and at least one point-of-care professional from each health care profession affected by the changes. Members were identified based on their expertise and willingness to champion the changes to their colleagues. Ideally, service users and their carers would have been included in design working groups, but at the time of implementation, the organization had neither a structured process in place to recruit service users or carers nor a strategy to ensure meaningful participation of service users and carers.

An eight-step framework based on project management principles [14] was developed for the implementation of a CPG for schizophrenia. The goals of the project were to leverage the electronic medical record (EMR) to integrate CPGs consistently into practice; involve point of care staff in the development of new processes; ensure that mechanisms were in place to monitor adherence to guideline recommendations; and realign existing resources to meet the guideline-derived clinical service offerings with no additional funding in order to achieve long-term sustainability in an environment of cost constraint.

### Step 1: clinical practice guideline selection

The internationally-recognized Appraisal Guidelines for Research and Evaluation (AGREE) instrument consists of 23 items to assess the quality of a CPG based on six domains: scope and purpose; stakeholder involvement; rigor of development; clarity and presentation; applicability; and editorial independence [15]. Gaebel and colleagues [16] reviewed and assessed five CPGs for the assessment and treatment of schizophrenia using the AGREE tool [The Royal Australian and New Zealand College of Psychiatrists; American Psychiatric Association; The German Association of Psychiatry, Psychotherapy and Psychosomatics; National Institute for Health and Care Excellence (NICE), Department of Health, United Kingdom; and The Schizophrenia Patient Outcomes Research Team, National Institute of Mental Health, United States]. Although the Canadian Psychiatric Association’s guideline was not included in the appraisal since the 1998 version scored low in a previous review [17], we considered the updated Canadian CPG [18], which was scored by a working group of local clinicians. The CPG that AGREE ranked highest overall was selected for implementation at Ontario Shores.

### Step 2: algorithm development

The selected guideline was reviewed and an algorithm was created for the assessment and treatment of schizophrenia for hospitalized service users, specifically and exclusively for the CPG. The work of distilling the full guideline into a succinct clinical algorithm was completed by the Physician Advisory Group to ensure that the new processes would be feasible with their workflow. The algorithm was built to cover all aspects of care from admission to discharge, including interventions for service users and their carers, as per the CPG.

### Step 3: gap analysis

A team of psychiatrists, interprofessional clinicians and informatics personnel conducted a gap analysis by creating a flowchart of our current state of treatment for persons with schizophrenia and comparing it with NICE-derived future-state processes depicted by the algorithm. Current processes that were not in accordance with the CPG were identified and a plan was made to operationalize missing assessment and treatment practices.

### Step 4: create governance structure

**Fig. 1:**
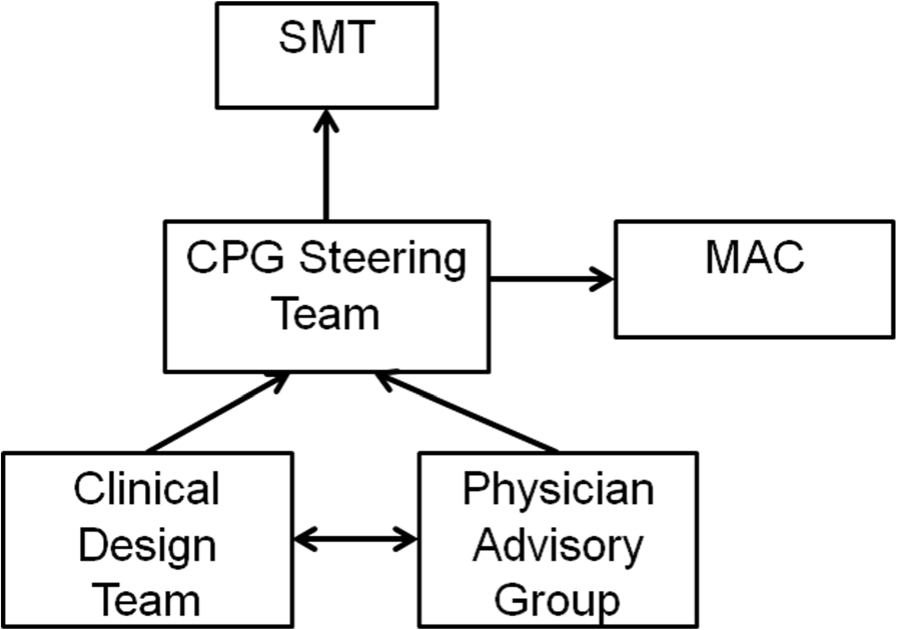
Governance structure developed for clinical practice guideline implementation. CPG, Clinical Practice Guideline; MAC, Medical Advisory Committee; SMT, Senior Management Team

A governance structure was developed to support CPG implementation (Fig. 1). This project was initiated as an outcome of senior management’s strategic plan, which was approved by Ontario Shores’ Board of Directors. Interprofessional design working groups reported to a steering committee, which was sponsored by the Vice-President of Medical Affairs and the Chief Nursing Officer; co-led by the Medical Director of Clinical Informatics (physician lead) and the Director of Professional Practice; and was composed of the Directors of Human Resources, Clinical Information, Project Management Office and Decision support, the administrative director of an inpatient unit, the Managers of Communications, Clinical Information and Pharmacy, the Clinical Manager of the Integrated Community Access Program and a Clinical Nurse Specialist from an inpatient unit. The physician lead was co-chair of the steering committee in addition to chair of the Physician Advisory Group and a member of the Medical Advisory Committee. Appropriate stakeholders were embedded into the governance structure to mitigate risk and to encourage ownership for the success of the initiative. Assumptions and constraints analysis was performed by the steering committee to validate assumptions and identify constraints and risk.

### Step 5: selection of key process adherence and outcome measures

The Physician Advisory Group used a modified Delphi Process to select key process adherence and outcome measures. The Physician lead developed a list of all possible adherence measures captured in the EMR and conducted a literature search for clinical outcomes in schizophrenia. This step was designed for inclusiveness, so all common outcome scales and applicable measures were included. The team reviewed the filtered list and discussed feasibility and Donabedian classifications [19] of each potential measure. Each member of the group independently scored each metric according to the five National Quality Forum criteria: importance, scientific acceptability, feasibility, usability, and related and competing measures [20]. Each was classified as being of high, medium or low importance based not only on their ability to indicate change in symptomatology, but recovery (e.g. improvements in relationships, level of functioning, ability to formulate and achieve goals). Mean scores for each domain were presented to the group. Low scoring metrics with limited feasibility or competing measures were discussed and rescored if necessary. The one outcome and four adherence measures that ranked highest were chosen as the primary quality assurance metrics, which populate a clinical dashboard that would be sent to every physician and clinical leader in the organization.

### Step 6: project charter development

A project charter, created by the physician lead included the scope of the project, accountabilities, executive sponsorship, timelines, budget and escalation strategies.

### Step 7: utilization of informatics

Informatics was included in all planning activities from the onset of the project to ensure that technology was leveraged to enable successful CPG implementation. Current and future-state work-flows were documented using process maps. Electronic clinical documentation tools for physicians, nurses and allied healthcare staff were revised in accordance with the findings of the gap analysis. Order sets and clinical panels (i.e. groups of tests routinely ordered to determine health status – for example, lipid panel for metabolic monitoring) were revised to align with the CPG. Extensive clinical decision support was embedded within the EMR to promote CPG adherence by building rules within the system whereby certain interventions and orders were automatically suggested when clinicians documented certain items within their templates. Online tools were developed for staff education to increase awareness of the CPG and aid in the transition.

### Step 8: realignment of therapeutic services

Decision documents for service realignment were created by clinical working groups to outline options for resource re-allocation for the purposes of operationalizing the guideline. Decision documents detailed the pros and cons of various options for operationalizing each recommendation, with consideration including resource use, cost, degree of clinical restructuring, local expertise, training needs and potential consequences to service users (i.e. clinical outcome, loss of service not included in the CPG, etc.). These were presented to senior management for final selection and approval. In order for this process to remain cost-neutral, the hiring of new personnel was out of scope for this project. Therapeutic Recreation was the discipline most affected by the clinical service delivery changes with CPG-recommended treatments replacing non-CPG activities. All training occurred during normal work hours to maintain neutral cost.

### Dissemination

**Fig. 2:**
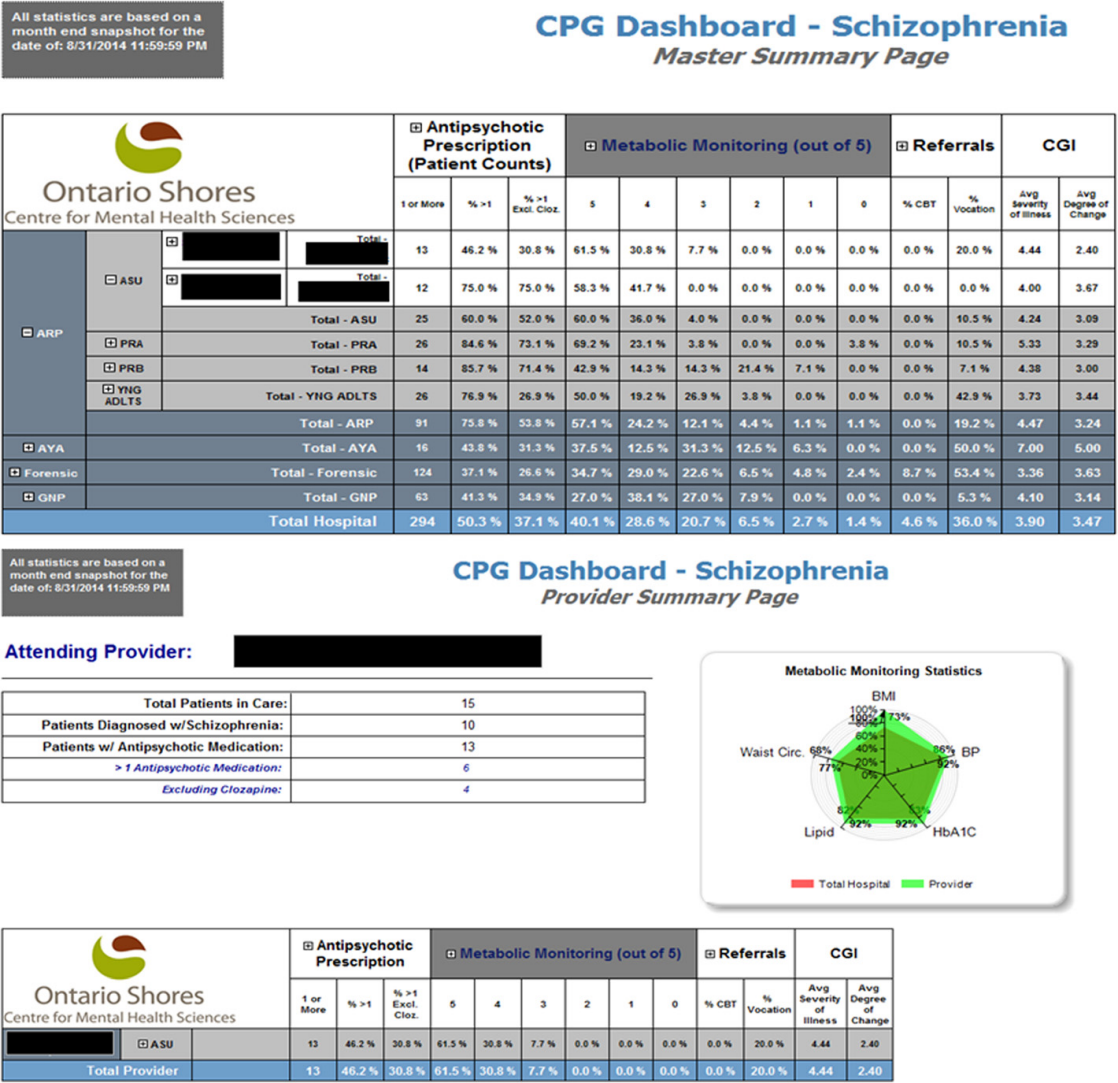
Clinical score card developed to provide clinicians with feedback regarding adherence to guidelines. ASU, PRA, PRB, YNG ADLTS, ARP, AYA, Forensic, GNP represent different units at Ontario Shores. BMI, body mass index; BP, blood pressure; CBT, cognitive behavioral therapy; CGI, Clinical Global Index; Cloz., Clozapine; HbA1c, glycated hemoglobin

Clinical dashboards were developed for physicians, clinical managers, clinical nurse specialists and administrative directors. These were compiled in the EMR and pushed out monthly for each physician and program to view their adherence to the prioritized metrics and compare them to hospital averages and other units (Fig. 2). They could not see the adherence of individual physicians. This dissemination strategy commenced May 2014 (i.e. following the first month of implementation) and continued throughout the follow-up period.

### Data processing

To ensure sustainability, all data processing was completed through data contained within the EMR; hence, all variables had to be accessible in the EMR. Adherence data was examined for the key process adherence metrics identified by the modified Delphi Process. Baseline was calculated from March 2014 (the month prior to implementation of the CPG) and monthly adherence was calculated for the 12 months following implementation (April 2014-March 2015). Adherence to antipsychotic polypharmacy guidelines was calculated as the percentage of service users, at the end of each month, prescribed only one antipsychotic (or any antipsychotic medication in combination with clozapine as per the NICE CPG [21]). Adherence to metabolic monitoring was calculated as the percent of metabolic risk appraisal measures (waist circumference, body mass index and blood pressure every 30 days; glycated hemoglobin lipid panel annually) monitored at the recommended intervals. Adherence to CBT-P referral was calculated as the percent of service users with schizophrenia who had been referred for CBT-P. Adherence to referral for supported employment was estimated as the percent of service users who had been referred to the vocational services, which served as a proxy measure. Referral was selected as a proxy rather than attendance of CBT-P or vocational rehabilitation services as referrals were completed through the EMR, while attendance was not captured as a discrete data component and was therefore not easily accessible for tracking. Additionally, since the adherence of interest was that of the clinician, not the service users, it was decided that referral would reflect this best. Descriptive analysis of the change in monthly summary data was completed. Data are presented as percent CPG adherence, unless otherwise stated.

## Results

### AGREE analysis

Based on the CPG review completed by Gaebel et al. [16] and the review of the Canadian Psychiatric Association guidelines completed by Ontario Shores’ clinicians, the NICE CPG for assessment and treatment of schizophrenia [21] was selected, as it was the highest ranked CPG according to the AGREE analysis with a total score of 90 [16].

### Gap analysis

The three identified clinical service gaps were lack of standardized art therapy, lack of carer assessment, and low capacity for CBT-P. Important opportunities for documentation standardization and the building of automated decision-support were also identified, but other major CPG treatment recommendations such as smoking cessation, diet and exercise were already standard treatments at Ontario Shores. A tracking template for psychopharmacologic trials over the course of a service-user’s illness, automated decision-support tools to promote adherence to the recommended metabolic monitoring protocols and referrals to evidence-based non-pharmacologic treatments such as CBT-P and vocational services, exception handling requiring physicians to enter a “reason” when prescribing multiple antipsychotics, and links to educational pamphlets for service-users were created.

### Process adherence and outcome measures

The modified Delphi process identified the Clinical Global Impression Scale for Schizophrenia as the priority metric for quantifying service user outcomes (Table 1). Four process adherence measures were identified, with percentage of service users on multiple antipsychotics (excluding clozapine as the second antipsychotic) ranked as number one, followed by percentage of service users with all five metabolic risk factors monitored at recommended intervals, percentage of service users who had been referred for supported employment/vocational services, and percentage of service users who had been referred to CBT-P.

**Table 1:** Service user outcome and process adherence metric selection via modified Delphi process

Outcome Measures	Importance	Validity	Scientific soundness	Usability	Feasibility	Ranking
CGI-SCH	HIGH	HIGH	MED	MED	HIGH	1
Degree of involvement in vocational rehabilitation	HIGH	HIGH	MED	MED	HIGH	
Brief Psychiatric Rating Scale	MED	HIGH	MED	MED	MED	
Degree of Independent Living	HIGH	HIGH	MED	LOW	MED	
PANSS	MED	MED	MED	MED	LOW	
Service user Satisfaction Survey Responses	HIGH	LOW	LOW	MED	MED	
Development of Metabolic Syndrome/Diabetes	HIGH	HIGH	MED	MED	MED	
GAF Score	MED	MED	MED	MED	MED	
Process Adherence Measures						
Percentage of Service users on Multiple Antipsychotics (excluding clozapine)	MED	HIGH	MED	HIGH	MED	1
Percentage of service users who have metabolic monitoring at recommended intervals	HIGH	HIGH	HIGH	HIGH	LOW	2
Percentage of service users referred for supported employment/vocational services	HIGH	MED	HIGH	HIGH	HIGH	3
Percentage of service users referred to CBT for psychosis	HIGH	MED	HIGH	HIGH	HIGH	4
Percentage of service users on recommended dosages (within therapeutic range) of antipsychotic medication	MED	MED	MED	HIGH	LOW	
Percentage of service users with a summary of previous medication trials	MED	MED	MED	HIGH	MED	
Percentage of first-episode service users who have had a MRI brain	MED	LOW	LOW	HIGH	HIGH	
Percentage of service users that have an annual physical exam	MED	LOW	LOW	HIGH	HIGH	

### Realignment of services

NICE guidance recommends offering 1:1 CBT-P for all service users with schizophrenia. Although this was one of the recommendations in the prepared decision document, it was not feasible to implement this in a cost-neutral way. Resources were realigned to ensure that referral to group CBT-P was available for all service users with schizophrenia and that 1:1 CBT-P could be offered if a service user did not respond to or was not suitable for the group format. Specifically, a CBT-P manual was chosen, a number of psychologists were trained in the provision of CBT-P, supervision groups were implemented, and decision-support was added such that a referral to CBT-P was suggested every time an admitting physician documented that a service user had schizophrenia as a primary diagnosis. Only referral to group CBT-P was captured in the EMR data. Clinicians indicated that they believed many service users were too ill or disorganized at the time of admission to benefit from a CBT-P group and as a result, often de-selected the recommended order that was triggered at admission. Standardized art therapy, including the modalities of music, visual art and drama/dance therapy, is recommended in the NICE guideline as an important intervention for service users with schizophrenia to reduce negative symptoms [21]. A decision document was created outlining various options for implementing standardized art therapy modules. Prior to CPG implementation, music and art were already included as therapeutic recreation activities. Since training and clinical supervision resources were already secured, music therapy was formalized according to the CPG. Art continued to be used as a therapeutic modality, but not in a formalized program.

**Fig. 3:**
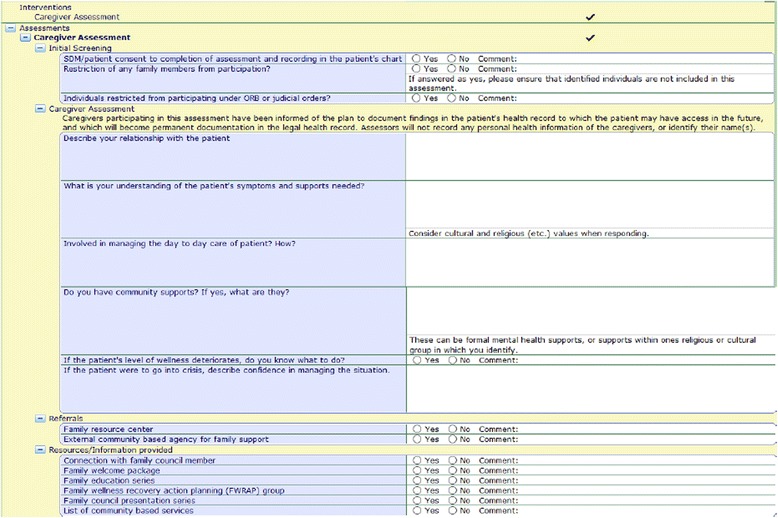
Screen shot of the carer assessment developed for social workers to complete with carers in order to comply with guideline

Carer support is recommended as an intervention in the treatment of schizophrenia. Verbal consent by service users (documented in the EMR) was required for carer involvement in care. In order to conform to the NICE CPG, enhancements were added to the existing processes. A standardized template was created in the EMR for a carer assessment to be completed by social workers during their work with carers (Fig. 3). Additionally, a number of standard carer support group interventions were made available and referral to the Family Resource Centre was prioritized.

Ontario Shores had already implemented a robust supported employment program and it was identified that additional resources were not likely required at the time of CPG implementation. Hence, it was decided that adherence to vocational services referral would be tracked and processes to improve adherence would be implemented if needed.

### Adherence data

**Fig. 4:**
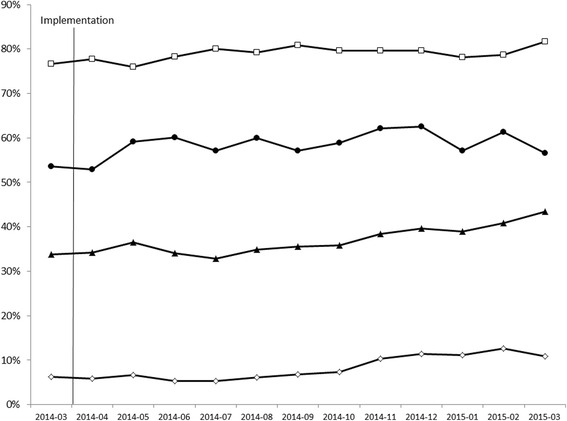
Clinical practice guideline adherence from baseline (March 2014) through 12-month follow-up. Closed *circles*, monotherapy; open *squares*, metabolic monitoring; closed *triangles*, vocational rehabilitation referral; open *diamonds*; cognitive behavioural therapy for psychosis referral

CPG adherence data from the month prior to implementation (March 2014) and the 12 months following implementation (April 2014-March 2015) are shown in Fig. 4. In March 2014, 53.4 % of service users were prescribed monotherapy (range across units 18.7–100 %), which increased to 62.7 % in November 2014 (range across units 27.3–100 %) and decreased back toward baseline by March 2015 (55.1 %; range across units 22.7–100 %), showing an overall 5.8 % increase over the follow-up period. In March 2014, 76.7 % of metabolic syndrome risk factors were monitored at the recommended intervals (range across units 60.0–90.0 %). There was a 6.3 % increase over the 12 month follow-up to 81.6 % adherence in March 2015 (range across units 0–100 %: note that the unit with 0 % had only one service user who met inclusion criteria). Referrals to CBT-P increased 74.8 % from 6.5 % (range across units 0–27.3 %) in March 2014 to 11.4 % (range across units 0–23.1 %) in March 2015. The percentage of service users referred to vocational rehabilitation increased 34.2 % from 36.6 % (range across units 0–81.3 %) in March 2014 to 49.1 % (range across units 0–76.2 %) in March 2015.

## Discussion

This paper describes the framework developed for the implementation of the NICE CPG for schizophrenia at Ontario Shores and describes clinician CPG adherence over the 12 months following implementation. Strong evidence regarding the implementation of CPGs in specialist mental health care is scant, [22] but literature from general mental health care supports systematic implementation of guidelines rather than passive dissemination of information [13, 23]. CPGs were more successfully implemented in the presence of strong professional support, peer review or quality circles [23, 24], with no increased or unmet costs, systems in place for tracking implementation, and clear guidance which reflected the clinical context [24]. Organizational support and culture [11, 12, 24] and computerized clinical decision support [4, 23, 24] have strong support as important facilitators of successful guideline implementation. Therefore, the alignment of practices at Ontario Shores with the NICE CPG for schizophrenia was undertaken as an organization-wide, cost-neutral initiative, involving senior management, interprofessional clinical leaders, working groups composed of point of care staff, and an informatics team. Informatics was highly involved and workflows and decision support tools were modified or created to conform to the CPG. Additionally, customized clinical dashboards were developed to provide feedback to clinicians regarding their adherence individually and in comparison to other practitioners and units. Finally, the CPG algorithms were posted on all clinical units and learning modules were created for the internal staff education site to promote adoption.

One of the goals of this project was to modify prescribing practices to reduce antipsychotic polypharmacy, which has been associated with increased side effects with little evidence to support substantial benefit [25]. In one study, the majority of service users who were switched to monotherapy showed no differences in PANSS scores or number of hospitalizations during the 6-month follow-up period compared to those prescribed combinations of antipsychotics [26], suggesting no additional benefit to polypharmacy. Nevertheless, polypharmacy remains prevalent worldwide, with 40–65 % of service users prescribed multiple antipsychotics [27–30]. Appropriate implementation of guidance has been shown to decrease the rate of antipsychotic polypharmacy prescription from 57 % at baseline to 16 % at 6-year follow-up [31]. In the present study, monotherapy rates were increased from 53.4 to 62.7 %, but this tended to return toward baseline over the following months. These prescribing practices are in line with international averages [27–30]. Ontario Shores is a tertiary-care mental health facility with a high proportion of treatment-refractory service-users. Notwithstanding their engagement with the CPG implementation, psychiatrists often noted that they did not want to risk clinical decompensation in some of their complex service users by switching from antipsychotic polypharmacy to monotherapy. The largest decrease in polypharmacy (i.e. increased adherence to guideline) occurred between April and May 2014 (polypharmacy decreased from 46.1 to 37.3 %), which is when the clinical dashboard was first pushed out to physicians, suggesting that awareness resulted in improved prescribing practices. From the current data, we are unable to determine why further improvements were not made or sustained. Future studies need to examine reasons for polypharmacy in order to create targeted strategies for reduction.

Metabolic abnormalities, which increase the risk of developing cardiovascular diseases and type 2 diabetes mellitus [32, 33], are common side effects of second generation antipsychotic therapy. Since the rate of cardiovascular mortality in men with schizophrenia is more than double that of the general population [34, 35], monitoring and subsequent management of cardiometabolic risk factors is an important component to schizophrenia treatment. A systematic review showed that, overall, adherence to metabolic monitoring guidelines in a population of service users on antipsychotic therapy was inadequate to suboptimal, with monitoring of individual risk factors ranging from 16 to 69 % [6]. In a cluster randomized feasibility trial, implementation of cardiovascular risk monitoring with a nurse-led intervention resulted in monitoring of some risk factors in approximately two thirds of service users compared to approximately one third in the control group, which received an education pack only with no specific leadership [7]. Determination of overall cardiometabolic risk, however, requires monitoring of all five components of the metabolic syndrome in each individual. Automated computer reminder systems increased monitoring of all five metabolic risk factors in an outpatient setting from 5 to 15 % overall [4], but performance evaluation feedback was not generated for clinicians. In the present study, 76.7 % of the metabolic risk factors were monitored at the appropriate intervals prior to guideline implementation and adherence increased slightly to 81.6 % at 12 months follow-up. Importantly, the percentage of service users who had all five metabolic risk factors monitored increased from 36.0 % prior to CPG implementation to 56.3 % at 12-month follow-up (data not shown). There was an increase in adherence when data became available at the clinician level and was disseminated. This feedback appears to be an important component of adherence to metabolic monitoring. The success of this initiative was also likely a result of clinical decision support, which included automated reminders for metabolic monitoring via an order set which was triggered anytime a physician indicated that a service user was being prescribed or about to be prescribed an antipsychotic.

According to the NICE guideline, CBT-P is a recommended treatment for schizophrenia. In practice, referral to CBT-P is generally low, though there is substantial variation across facilities. Reported CBT-P referral rates range from 6.9 to 72 % [36–39], though the actual number of service users receiving the intervention was lower. Implementation of the NICE CPG advising CBT-P at Ontario Shores resulted in a referral rate of only around 7 %, which remained low increasing to only 11.4 % over the follow-up period. Berry and Haddock [40] identified a number of barriers to CBT-P implementation, which tended to cluster into three main categories: 1) capacity (e.g. level and quality of training and supervision); 2) how the service was configured (e.g. caseload size, responsibilities of care teams, managerial priorities); and 3) service user and carer barriers (e.g. a lack of desire for psychological interventions or preferring other types of intervention). Many of these barriers were anticipated in the development of the implementation process at Ontario Shores, but efforts to mitigate barriers were not successful and adherence remained low. Adherence to this guideline may be improved by developing automated reflexive orders at later points in the hospitalization rather than just on admission. It is important to note that resources were not a limiting factor in the availability of CBT-P in the group format so additional education, analyses and decision-support strategies should result in improved utilization of CBT-P. Since Ontario Shores was unable to implement 1:1 CBT-P for all service users as per the guidelines, the effectiveness of group CBT for these service users should be evaluated to assess whether this intervention improves outcomes.

Supported employment programs have proven to be superior to prevocational training for improving the likelihood that people with mental illness will attain competitive employment [41, 42]. Ontario Shores already had a robust supported employment program in place for service users as well as other vocational services for those who are not ready for supported employment. Vocational counselors work in partnership with the Ontario Disability Support Program to assist service users in finding work within the community. Automated referrals to vocational therapy via a suggested order are triggered when a physician enters schizophrenia as the primary diagnosis at admission. Vocational therapy performs an assessment on each service user referred with the goal of providing supported employment when clinically appropriate. Similarly to CBT-P, admitting physicians can deselect the suggested referral if they believe the service-user would not benefit from the vocational therapy assessment at the time of admission. More analysis is required to determine if all service users who would benefit are being offered supported employment.

### Limitations

This paper describes Ontario Shores’ implementation of the NICE CPGs for schizophrenia [21]. Some limitations should be noted. The adherence data should be interpreted with caution. Adherence numbers presented indicate only the overall percentage of completed/referred tasks and do not take into account reasons for non-adherence, which could include not clinically indicated or service user/carer refusal. Reasons for non-adherence to guidelines should be documented and reported in the future. Additionally, adherence rates may be an artifact of improved documentation rather than increased adherence to the CPG *per se*. The reported variation in adherence measures (i.e. range across units) should be interpreted with caution as qualifying service users (i.e. those diagnosed with schizophrenia or schizoaffective disorder) ranged from 1 to 25 per unit over the 13 months of data collection (those with 0 qualifying service users were not included).

The majority of individuals with schizophrenia are treated in the community or acute care hospitals; hence, the NICE CPG was developed based on evidence generated primarily in these settings. Ontario Shores is a specialty hospital providing services for complex and serious mental illnesses, so the recommendations are not appropriate for all of the services users treated at this facility. A recent systematic review noted that guidelines should be adapted locally to account for values, resources, context and feasibility [22]. The challenge for the future will be to consider these issues to outline appropriate adherence targets for each metric, while considering the unique composition of our service user population.

While the specific programs and services initiated and reported here may not be generalizable to all mental health facilities, the general framework and tools should be customizable to most circumstances. Our goal is to create a collaborative network and to foster a continuous cycle of learning to share experiences and develop a set of tools for successful implementation of CPGs based on learnings from numerous sites with varying levels of care. A toolkit and workbook have been developed, which provide access to the process used in implementation [43].

Throughout the implementation of CPGs at Ontario Shores many lessons were learned. It was important to have strong engagement with physicians and interprofessional clinicians; high levels of transparency and justification of processes was required to support physician buy-in. Moreover, physician behavior was not the only factor affecting adherence, as the interprofessional team and service user choices could also have affected the adherence score. Involvement of informatics to develop structured templates, order sets, clinical panels, decision-support and clinical scorecards was essential to promote adherence and to provide relevant feedback for improvement.

Future research is needed to investigate the effects of CPG implementation on relevant service user outcomes. Reasons for non-adherence need to be examined in order to develop targeted quality improvement initiatives to increase adherence. Additionally, proper follow-up procedures should be considered. In one study, despite better adherence to metabolic monitoring with a nurse-led implementation compared to education only, there was no difference in the referral to relevant behavioral interventions or prescription of indicated pharmacological treatments [7]. Although not measured in this study, it is necessary for clinicians to respond appropriately to reduce adverse metabolic sequelae if the monitoring results indicate increased risk.

## Conclusions

In conclusion, this paper describes a promising strategy to support the implementation of and adherence to CPGs in specialized mental health. This project is a starting point for collaborative network building, providing a framework and tool kit that can be customized to meet organizational needs.

## Abbreviations

AGREE, the appraisal guideline for research and evaluation; CBT-P, cognitive behavior therapy for psychosis; CPG, clinical practice guidelines; EMR, electronic medical record; NICE, National Institute for Health and Care Excellence; Ontario Shores, Ontario Shores Centre for Mental Health Sciences
